# Causal association between metformin and arrhythmias: A Mendelian randomization study

**DOI:** 10.1097/MD.0000000000046091

**Published:** 2026-01-09

**Authors:** Wen-Pei Qin, Jin-Yu Zhang, Qian Zhao, Fen Liu, Xiao-Mei Li, Yi-Ning Yang

**Affiliations:** a State Key Laboratory of Pathogenesis, Prevention and Treatment of High Incidence Diseases in Central Asia, Department of Cardiology, The First Affiliated Hospital of Xinjiang Medical University, Urumqi, China; b Clinical Laboratory, The First Afiliated Hospital of Xinjiang Medical University, Urumqi, China; c Rehabilitation Department, The First Affiliated Hospital of Xinjiang Medical University, Urumqi, China; d Xinjiang Key Laboratory of Cardiovascular Disease Research, Clinical Medical Research Institute The First Affiliated Hospital of Xinjiang Medical University, Urumqi, China; e Department of Cardiology, People’s Hospital of Xinjiang Uyghur Autonomous Region, Xinjiang, China; f Xinjiang Key Laboratory of Cardiovascular Homeostasis and Regeneration Research, Xinjiang, China.

**Keywords:** arrhythmia, mendelian randomization, metformin

## Abstract

There is ongoing discussion in the medical community on the effect of metformin in cardiac arrhythmias. Investigating the causal relationship between metformin and arrhythmias was the target of the research. We utilized the Mendelian randomization (MR)-based platform to collect genome-wide association data linking arrhythmias and metformin. We utilized SNPs connected to metformin as instrumental variables (IVs) to evaluate the causative link between metformin and arrhythmia through two-sample MR analysis. Four statistical techniques were employed: MR-Egger regression, weighted median estimator, weighted mode method, and inverse-variance weighted (IVW) method. The impact of individual SNPs on the outcomes of IVW analyses was investigated using the leave-one-out method, and the study’s possible bias was examined using a funnel plot to guarantee the results’ robustness. We identified 40 independent single nucleotide polymorphisms (SNPs) of genome-wide significance from GWAS data for metformin as IVs. The IVW method supported a causal relationship between metformin and reduced incidence of arrhythmia (OR = 0.0093; 95% CI = 0.002–0.055; *P* = 2.72E−07). MR-Egger regression indicated that there is no need to consider the effect of gene pleiotropy on the results of the study (intercept = 0.0014; *P* = .874). Although the MR-Egger method didn’t pinpoint a causal link, it did reveal a similar directionality in the β values (OR = 0.0056; 95% CI = 9.384E−06 to 3.369; *P* = .1207). Fortunately, the weighted median and weighted mode techniques demonstrated a causal link between metformin and a decreased arrhythmia risk (OR = 0.004; 95% CI = 0.0003–0.052, *P* = .000023; OR = 0.0025; 95% CI = 7.418E−05 to 0.085, *P* = .001915). Neither Cochran *Q* test nor the funnel plot showed signs of directional pleiotropy, heterogeneity, or asymmetry. The sensitivity of the method was analyzed by leave-one-out method, and the results suggested that the method was stable. With the MR method, we’ve substantiated a potential cause-and-effect link between metformin usage and cardiac arrhythmia.

## 1. Introduction

Arrhythmia is a prevalent cardiovascular condition affecting individuals across all age groups, with increased prevalence in older adults. Atrial fibrillation (AF), the most severe form of arrhythmia,^[1]^ has become a major public health issue. It is estimated that 33.5 million persons all over the world experienced AF by 2010.^[2]^ Projections indicate that the amount of AF cases in the Americas is expected to increase to 15.9 million until 2050,^[3]^ and in the Europe to 17.9 million by 2060.^[4]^ The rising incidence and prevalence, coupled with a high lifetime risk, make AF a critical disease in the population, characterized by high morbidity, mortality, and healthcare costs.^[5]^ Ventricular arrhythmias, though less common than AF, are often the last arrhythmias before sudden cardiac death.^[6]^ However, the lack of efficacy and negative impacts of the present antiarrhythmic medications restrict their applicability.^[7]^ Therefore, it is imperative to seek alternative solutions to mitigate the high incidence and societal burden.

Metformin has been used in the treatment of diabetes for 60 years and currently serves as a first-line treatment in diabetes. Actually, the underlying mechanisms of its therapeutic action are complex and not fully grasped.^[8]^ Some recent studies have showed a decrease to the prevalence of AF^[9–12]^ and a lower risk of ventricular arrhythmia,^[9,13,14]^ However, metformin failed to show antiarrhythmic benefits in other studies.^[15–17]^ Most clinical evidence for metformin’s antiarrhythmic effects is derived from research using observations, which unmeasured confounding factors may influence. Conventional randomized controlled trials (RCT) face challenges related to cost, duration and ethical considerations.

Mendelian randomization (MR) is a strategy which utilizes genetic variants as instrumental variables (IVs) for exposure factors to examine whether an observed association between a danger factor and an outcome implies a causal connection. Genetic variants, when employed as IVs in accordance with Mendelian rules, reduce the likelihood of bias from confounding effects.^[18]^ Databases are from the open GWAS project and do not require ethical review. In the research, we employed a two-sample MR approach through GWAS information to investigate if metformin causally affects the incidence of arrhythmias.

## 2. Materials and methods

### 2.1. Data sources and selection of genetic

This investigation utilized single nucleotide polymorphisms (SNPs) connected to metformin as IVs to assess the causative link between metformin and arrhythmia through two-sample MR analysis utilizing the Open GWAS analysis database.^[19]^ This study employed a two-sample MR approach, relying on 3 fundamental assumptions: Firstly, the SNPs serving as IVs must be closely linked to the metformin (the exposure). Secondly, the chosen SNPs must be squeaky clean when it comes to confounding variables—completely independent of them. And lastly, the only way these IVs should influence arrhythmias (the outcome) is indirectly, through their link with metformin use (the exposure); there shouldn’t be any kind of direct pathway. To ensure the robustness of our analysis, we assessed heterogeneity and potential horizontal pleiotropy using Cochran *Q* test and Egger regression method. Furthermore, we conducted a sensitivity analysis to verify the reliability of our findings.

For this study, genetic variation data were sourced from the MR Base database (http://www.mrbase.org/). According to the GWAS catalogue of the National Human Genome Research Institute and European Bioinformatics Institute (NHGRI-EBI),^[20]^ metformin’s GWAS ID is ukb-b-14609. Arrhythmia data was sourced from the bbj-a-86 Open GWAS project (Table 1). No ethical clearance was necessary for this research due to the use of publicly accessible aggregate data. Every single study incorporated into this research was pre-approved by the respective Ethics Committees from the original research, and all participants willingly offered their written informed consent. Details of their ethical clearance are documented within the original scholarly articles.

**Table 1:** Source of the genome-wide association study data.

Exposure/Outcome	Database	Year	Author	Participants	Number of SNPs	Web Source if public
Metformin (ukb-b-14609)	UKB	2018	Ben Elsworth	462,933 individuals (11,552 use cases and 451,381 controls) of European ancestry	9,851,867	https://gwas.mrcieu.ac.uk/datasets/ukb-b-14609/
Arrhythmia (bbj-a-86)	BBJ	2019	Ishigaki K	212,453 individuals (17,861 use cases and 194,592 controls) of East Asian population	8,885,805	https://gwas.mrcieu.ac.uk/datasets/bbj-a-86/

SNPs = single nucleotide polymorphisms.

We set the statistical significance threshold at “*P* < 5 × 10^−8^; linkage disequilibrium *r*^2^ < 0.001” to lessen the impact of linkage disequilibrium and identify SNPs related to metformin. To harmonize the exposure and outcome data, we integrated SNPs associated with both, weeding out incompatible alleles and palindromic SNPs with intermediate allele frequencies along the way. We collected summary statistics for 40 SNPs connected to metformin as the IVs on metformin. We employed the publicly accessible statistical summary dataset from a GWAS published in 2019 in an open GWAS project called bbj-a-86 (total = 212,453; cases = 17,861, controls = 194,592) on the self-reporting of non-cancer illness codes: arrhythmias as the outcome.

### 2.2. Statistical analysis for MR

The primary causal effects were assessed using the inverse-variance weighted (IVW) technique, with further supporting evidence provided by weighted median, weighted mode, and MR-Egger regression method. The odds ratio (OR) and 95% confidence interval (CI) values were computed accordingly. *P*-values < .05 were considered statistically significant. The heterogeneity of IVs was analyzed using Cochran *Q* test.^[21]^ There is no substantial heterogeneity if *P* > .05. It can be presumed that there is no horizontal pleiotropy in MR-Egger regression if *P* > .05 and the intercept goes to 0. The impact of individual SNPs on the outcomes of IVW analyses was investigated using the leave-one-out method, and the study’s possible bias was examined using a funnel plot to guarantee the results’ robustness.^[22]^ Every MR analysis was carried out using the MR Base platform (App version: 1.4.3 8a77eb [October 25, 2020], R version: 4.0.3).

## 3. Results

### 3.1. IVs for MR

Taking metformin as the exposure factor, 40 genome-wide significance SNPs related with metformin were acquired as IVs based on the screening criteria (Table 2). In total, 20 of the 40 SNPs were Negatively linked with arrhythmia, although they were not statistically significant. The sum of the *r*^2^ statistic was equal to 0.54%, which represents the variance of exposure. This can be attributed to the genetic variants acting as IVs. The *F* values were all larger than 10, indicating that the instrumental variable had sufficient strength.

**Table 2:** Characteristics of the single nucleotide polymorphisms associated with metformin and their associations with arrhythmia.

SNPs	Chr	EA	EAF	Metformin			Arrhythmia		
				β	SE	*P*	β	SE	*P*
rs10001190	4	G	0.632112	0.0026373	0.000335655	3.90E−15	0.234504	0.193976	.226689
rs10195252	2	C	0.405055	−0.00191685	0.000329621	6.10E−09	−0.0127156	0.0211983	.548613
rs10420309	19	G	0.437532	−0.00189448	0.000327625	7.40E−09	−0.00295021	0.0138035	.830759
rs10965246	9	C	0.176686	−0.00429996	0.000424723	4.30E−24	0.0332367	0.0114476	.00369173
rs11257655	10	T	0.208209	0.00273499	0.000398227	6.50E−12	−0.0220116	0.0121271	.0695136
rs11658063	17	G	0.602828	−0.0026474	0.000333353	2.00E−15	0.0150027	0.0129921	.248193
rs11708067	3	G	0.242368	−0.00216544	0.000377254	9.50E−09	0.0901192	0.462386	.845471
rs1215468	13	G	0.291405	−0.00292874	0.00035782	2.70E−16	0.016387	0.0127101	.197299
rs13266634	8	T	0.309592	−0.00254288	0.000350136	3.80E−13	0.0140264	0.0115039	.222741
rs1421085	16	C	0.403451	0.00352358	0.000329648	1.10E−26	0.0111495	0.0140656	.427967
rs1496653	3	G	0.203441	−0.00291936	0.000401455	3.50E−13	0.014132	0.0155734	.364172
rs1515096	2	T	0.426595	0.00254389	0.000356342	9.40E−13	0.0267383	0.0197484	.175754
rs1613295	10	G	0.578289	0.00240145	0.000327761	2.40E−13	−0.0247358	0.0117918	.0359302
rs17036160	3	T	0.117453	−0.00313639	0.000503572	4.70E−10	0.0400284	0.0338506	.237007
rs17513135	1	T	0.227468	0.0022746	0.000385776	3.70E−09	0.00944655	0.016003	.55499
rs1800961	20	T	0.030964	0.00536609	0.000933475	9.00E−09	−0.0683172	0.0571587	.232001
rs2009222	19	C	0.632367	0.00189842	0.000334869	1.40E−08	0.00708988	0.0173006	.681948
rs2237895	11	C	0.415681	0.00249846	0.000327645	2.40E−14	−0.0293494	0.0117957	.012841
rs2796441	9	A	0.418465	−0.00182507	0.000327318	2.50E−08	0.00417357	0.0116398	.719923
rs34744311	10	T	0.377322	−0.00284808	0.00033433	1.60E−17	0.0184147	0.0140612	.190327
rs34872471	10	C	0.291831	0.0085635	0.000356221	1.10E−127	−0.0360739	0.0274576	.188912
rs459193	5	G	0.746628	0.00236399	0.000371114	1.90E−10	−0.0130201	0.0113183	.249997
rs4752792	11	A	0.544491	0.00209176	0.00032437	1.10E−10	−0.00231387	0.0128108	.856668
rs4932264	15	C	0.729643	−0.0022175	0.000364805	1.20E−09	0.00567841	0.0137957	.680627
rs67232546	11	T	0.212505	0.00227404	0.000398149	1.10E−08	−0.0109271	0.0173949	.529887
rs6769511	3	C	0.315782	0.00318225	0.000348036	6.00E−20	0.00222538	0.0119681	.85249
rs7018475	9	G	0.257503	0.00272318	0.00037	1.80E−13	−0.00106281	0.0115976	.926984
rs7177055	15	A	0.717474	0.00224939	0.000358865	3.70E−10	−0.0293359	0.0116408	.0117328
rs72802357	16	T	0.078087	−0.00404659	0.000606272	2.50E−11	−0.0409318	0.0456813	.370238
rs73188924	22	A	0.224797	0.00217376	0.000389733	2.40E−08	−0.0168993	0.0185602	.362554
rs7482891	11	G	0.622096	−0.00217361	0.000334189	7.80E−11	0.0269818	0.0211124	.201245
rs76550717	11	G	0.159023	−0.00278832	0.000444411	3.50E−10	−0.0407559	0.0266383	.126024
rs76675804	2	C	0.100098	−0.00418436	0.000539519	8.80E−15	−0.0474514	0.467415	.919139
rs7756992	6	G	0.266365	0.00319744	0.000365589	2.20E−18	−0.034211	0.0113036	.00247355
rs780093	2	C	0.615229	0.00206338	0.000332018	5.10E−10	−0.0177106	0.011445	.121755
rs849142	7	C	0.505093	−0.00239601	0.00032333	1.30E−13	0.189244	0.145015	.191896
rs947791	11	A	0.217618	0.00228897	0.000392464	5.50E−09	−0.0140448	0.0147585	.341277
rs9669278	12	C	0.51868	0.00189443	0.000325329	5.80E−09	−0.0318351	0.0178921	.0751934
rs987237	6	G	0.179582	0.00242652	0.0004213	8.40E−09	0.00739055	0.0137887	.591969
rs9957264	18	A	0.166478	−0.00262705	0.000435291	1.60E−09	0.00330345	0.0167991	.844106

SE = standard error, SNP = single nucleotide polymorphism.

### 3.2. Causal relationship between metformin and arrhythmia

**Figure 1.**
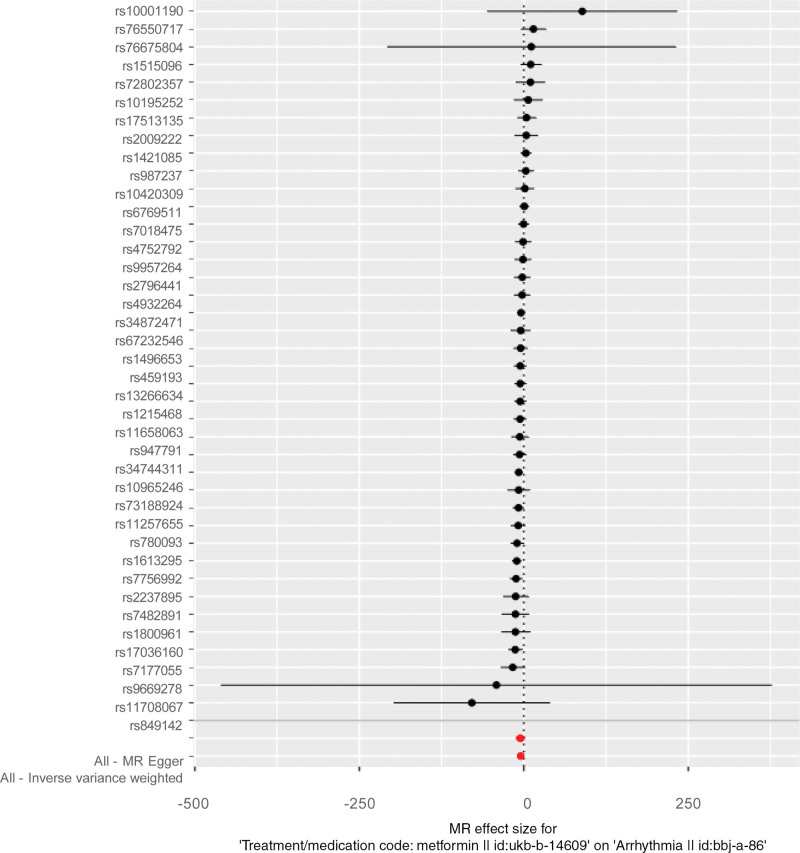
Forest plot depicting the impact of SNPs linked to metformin on arrhythmias; red lines indicate MR outcomes from the MR-Egger and IVW analyses. IVW = inverse-variance weighted, MR = Mendelian randomization, SNPs = single nucleotide polymorphisms.

**Figure 2.**
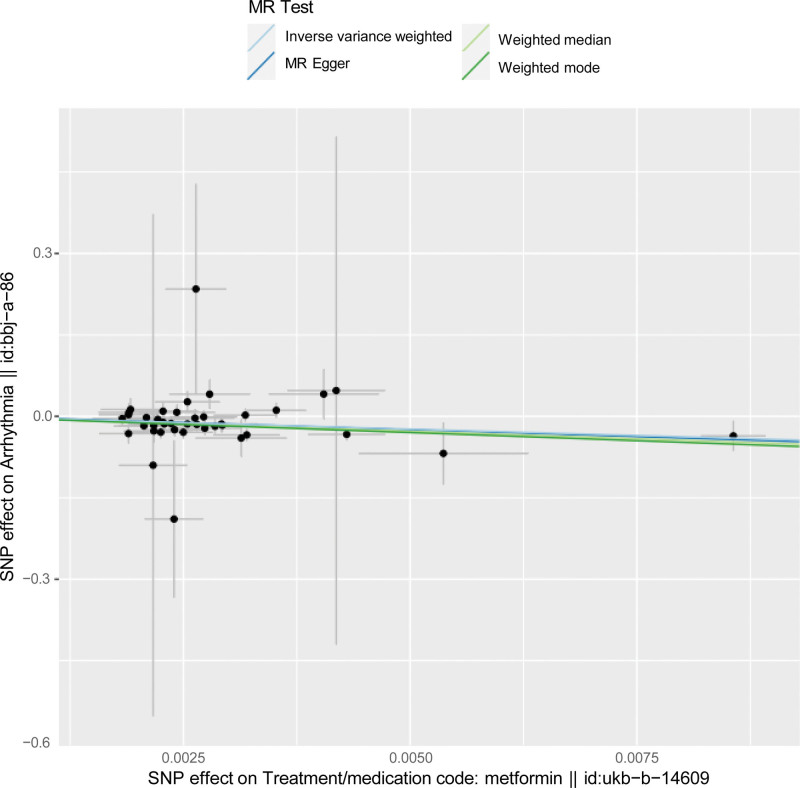
Scatter plots depicting the genetic associations with metformin versus those with arrhythmia are presented. The causal relationship for each method is denoted by the slope of its corresponding line. The deep blue line represents the MR-Egger estimate, the green line represents the weighted median estimate, and the blue line represents the IVW estimate. IVW = inverse-variance weighted, MR = Mendelian randomization.

The IVW techniques corroborated a causal link between metformin and the decreased incidence of arrhythmia (OR = 0.0093; 95% CI = 0.002–0.055; *P* = 2.72E−07; Table 3, Figs. 1 and 2). This study was checked for horizontal gene pleiotropy with MR-Egger regression analysis, and MR-Egger regression indicated that there is no need to consider the effect of gene pleiotropy on the results of the study (intercept = 0.0014; *P* = .874). Although the MR-Egger method didn’t pinpoint a causal link, it did reveal a similar directionality in the β values (OR = 0.0056; 95% CI = 9.384E−06 to 3.369; *P* = .1207). Fortunately, the weighted median and weighted mode techniques demonstrated a causal link between metformin and a decreased arrhythmia risk (OR = 0.004; 95% CI = 0.0003–0.052, *P* = .000023; OR = 0.0025; 95% CI = 7.418E−05 to 0.085, *P* = .001915; Table 3, Fig. 2). The results using the MR-Egger method are different from several other methods. The study’s findings may suggest a possible causal relationship between metformin and the reduction of arrhythmia risk, given that the IVW maintains better precision in the estimations than the MR-Egger analysis.

**Table 3:** Mendelian randomization results of the causal association between metformin and arrhythmia using 4 methods.

MR method	Number of SNPs	Beta	SE	OR	95% confidence interval	Association *P*‐value	Cochran *Q* statistic	*I* ^2^*^^	Heterogeneity *P*‐value
Inverse-variance weighted	40	−4.682	0.9106	0.0093	0.002 to 0.055	2.72E−07	41.98	0.095	.302
MR-Egger	40	−5.181	3.263	0.0056	9.384E−06 to 3.369	.1207	42.01	0.072	.342
Weighted median	40	−5.515	1.303	0.004	0.0003 to 0.052	.000023			
Weighted mode	40	−5.985	1.798	0.0025	7.418E−05 to 0.085	.001915			

Beta = beta coefficient, MR = Mendelian randomization, OR = odds ratio, SE = standard error, SNP = single nucleotide polymorphism.

* *I*^2^ = (*Q* − df)/*Q*.

### 3.3. Bias analysis

**Figure 3.**
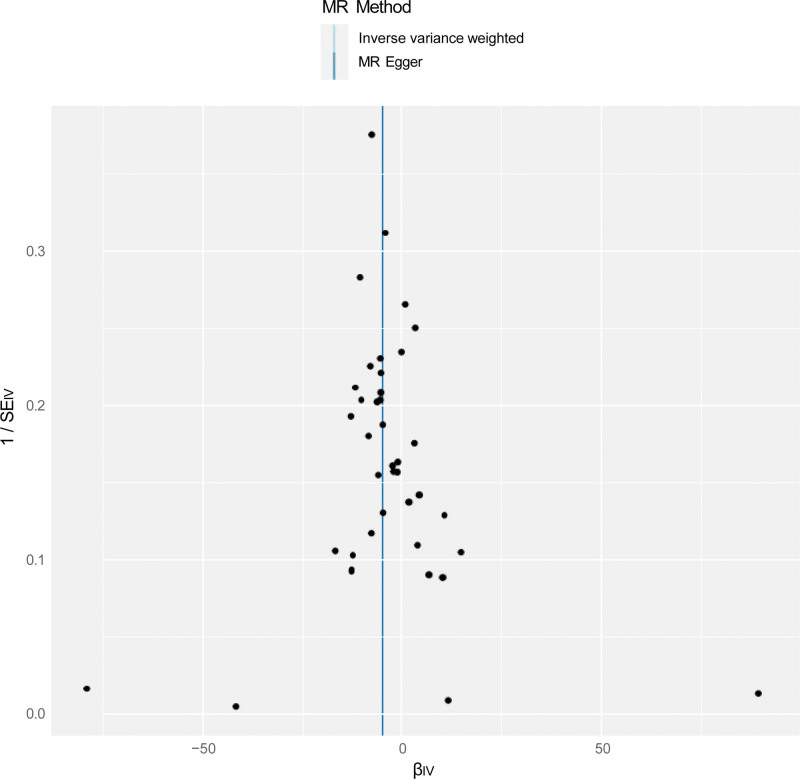
Funnel plot to assess heterogeneity. The MR-Egger estimate is shown by the deep blue line, while IVW estimate is represented by the blue line. IVW = inverse-variance weighted, MR = Mendelian randomization.

The funnel plots assessment demonstrated general symmetry, suggesting that there isn’t clear bias for the influence in the findings (Fig. 3).

### 3.4. Heterogeneity and sensitivity test

The *P* > .05 of the *Q* test statistic indicates no statistical significance. there was no discernible variation across the IVs (*P* > .05, Table 3). MR-Egger regression revealed that the intercepts were near to zero, with *P* > .05. There is no need to consider the effect of gene pleiotropy on the results.

**Figure 4.**
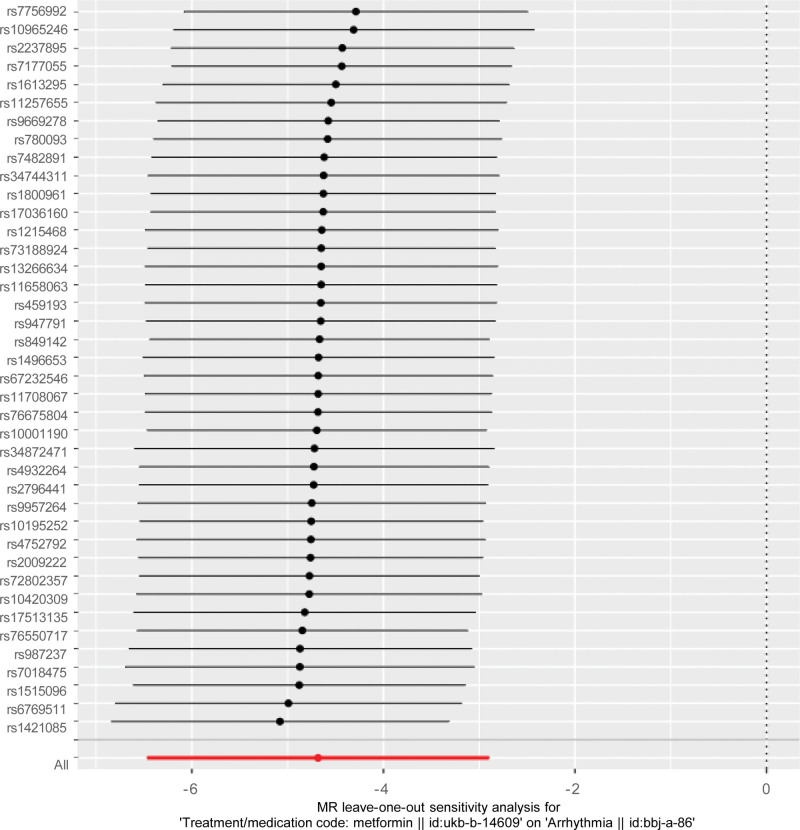
Forest plots of leave-one-out analysis. The placements of the red marks are below zero. The black dots appear on the left side of the ineffective line. This shows that eliminating any of the SNPs won’t make a significant difference to the result. SNPs = single nucleotide polymorphisms.

Sensitivity assessment utilizing the leave-one-out technique to evaluate the stability of the MR results by eliminating each SNP in turn and using the IVW approach on the other SNPs. The stability and trustworthiness of the current study’s findings were demonstrated by the fact that no significant connections were discovered following the removal of any SNPs and that the majority of the results were near the total effect values, which had no discernible impact on the findings (Fig. 4).

## 4. Discussion

Metformin is used by over 150 million people worldwide, the great majority of whom use it to treat type 2 diabetes.^[23]^ The therapeutic profile has also broadened as we gain a better grasp of novel mechanisms.^[24]^ The impact of metformin on arrhythmias has consistently been a contentious issue in the medical community.^[25]^ Determining whether metformin can also reduce the incidence of arrhythmias in nondiabetic and using it as a cardiovascular drug still requires more rigorous RCT methods. RCT investigations, however, require time, money, and ethical scrutiny. Despite having less evidence than RCT, MR analysis has been employed extensively in academic research and is not constrained by experimental or ethical requirements. In comparison to observational research, it is also less vulnerable to reverse causality and possible confounders.^[26]^ Consequently, MR analysis is regarded as a natural RCT study, and its findings are reliable.^[27,28]^

In this research, we looked into the relationship between metformin and lowering incidence of arrhythmias using a two-sample design. According to our findings, there might be a causal association between metformin and a lower incidence of arrhythmias. We used 4 distinct estimating techniques to assess the relationship between them. The IVW, weighted median and weighted mode analysis supported a causal association between metformin and a lower incidence of arrhythmias, despite the inconsistency of the 4 distinct estimating techniques. Knowing that IVW method and the weighted median estimator retains more precision in estimates than the MR-Egger analysis,^[29]^ this MR study suggested that metformin may have a causal role in the risk reduction of arrhythmias. This is consistent with the results of some observational studies,^[30–32]^ which have found an association between metformin and risk reduction of arrhythmias. The recent results may give more chances to further understand the processes potential metformin’s benefits on arrhythmia risk reduction. The link between metformin and a lower incidence of arrhythmias does not seem to be due to improved glycemic management, but rather a standalone antiarrhythmic activity.^[33]^ The possible mechanism of metformin’s antiarrhythmic effect is the alleviation of inflammation, reducing levels of pro-inflammatory cytokines,^[34,35]^ Reducing oxidative damage,^[30]^ promoting mitochondrial oxidative phosphorylation,^[36]^ reversing remodeling,^[37]^ promoting cardiac fatty acid oxidation^[38]^ and maintaining ATP levels in ischemic myocardium.^[39,40]^ Li et al^[41]^ used the MR to investigate the causal link between metformin treatment and the incidence of AF. Their findings indicated that there was no discernible causal connection between metformin and the incidence of AF. Our study reveals a broader range of arrhythmias, transcending mere AF, potentially accounting for the divergent results.

There are several limitations to the current investigation. First, exposure data in this study originated from European subjects, while outcome data derived from East Asian individuals. Population stratification bias is a potential concern, though our results demonstrate robustness, and it was unclear if the results can be directly transferred to other groups, necessitating further analysis. Second, two-sample MR method might exaggerate the connection between SNP and exposure due to over-identification.^[42]^ Third, The types of arrhythmias included in the study data were not clearly described, so the outcome events were relatively ambiguous, and it was not possible to determine which arrhythmias were affected.

In conclusion, In order to better understand how metformin affects arrhythmias, our study analyzed 40 SNPs linked to the metformin. The results indicated that metformin might be causally connected with a decreased risk incidence of arrhythmias. Further investigations are required to find out the underlying mechanisms of this causal relationship.

## Acknowledgments

We deeply thank the GWAS website for its supplied data.

## Author contributions

**Conceptualization:** Yi-Ning Yang.

**Data curation:** Wen-Pei Qin.

**Formal analysis:** Wen-Pei Qin, Jin-Yu Zhang, Qian Zhao, Fen Liu, Xiao-Mei Li, Yi-Ning Yang.

**Investigation:** Qian Zhao.

**Methodology:** Jin-Yu Zhang, Fen Liu, Xiao-Mei Li, Yi-Ning Yang.

**Writing – original draft:** Wen-Pei Qin.

**Writing – review & editing:** Jin-Yu Zhang, Qian Zhao, Fen Liu, Xiao-Mei Li.
